# Combined analysis of transcriptome and WGCNA reveals the mediating role of JA in the low male fertility of loquat H30-6

**DOI:** 10.3389/fpls.2025.1648313

**Published:** 2025-09-12

**Authors:** Junxiu Wang, Jinting Pan, Qingqing Xia, Yudie Duan, Danlong Jing, Jiangbo Dang, Qigao Guo

**Affiliations:** ^1^ Key Laboratory of Agricultural Biosafety and Green Production of Upper Yangtze River (Ministry of Education), Chongqing Key Laboratory of Forest Ecological Restoration and Utilization in the Three Gorges Reservoir Area, College of Horticulture and Landscape Architecture, Southwest University, Beibei, Chongqing, China; ^2^ Chongqing University of Education, Nan'an, Chongqing, China

**Keywords:** loquat, low male fertility, seedless fruit, transcriptome, WGCNA, jasmonic acid

## Abstract

The number of seeds is an important factor limiting the palatability and processing efficiency of loquat. Loquat H30-6 has been found to bear fruits with few seeds, which is attributed to its low male fertility. To investigate the molecular mechanism underlying the low male fertility of H30-6, in this study, RNA-seq was performed using pre-meiotic, meiotic and mature anthers from H30-6 and the other two loquat varieties with normal male fertility. The results indicated that a total of 331 differentially expressed genes (DEGs) were identified between H30-6 and the other two loquats and these DEGs were mainly enriched in pathways related to jasmonic acid (JA). WGCNA revealed that module ‘green’ were highly positively correlated with seed quantity (*r* = 0.46), suggesting that module ‘green’ is the candidate module. Enrichment analysis showed that genes in module ‘green’ were also primarily enriched in JA biosynthetic process, regulation of JA mediated signaling pathway and response to JA, implying that JA may play a key role in the molecular mechanism underlying the low male fertility of H30-6. The detection of UPLC-MS/MS indicated that the contents of JA and its derivatives in the flower bud of H30-6 were significantly lower than those in ‘Huabai 1’ (with normal male fertility). Besides, exogenous application of MeJA improved the pollen quantity per anther and seed numbers of H30-6. Our work indicates that the suppression of JA-related gene expression and the low JA content in immature flower buds may be key factors for H30-6 male sterility. These findings provide a theoretical basis for seedless breeding of loquats and lay the foundation for the development of stamens in fruit trees.

## 1 Introduction

Loquat (*Eriobotrya japonica* Lindl.) is a popular subtropical fruit of the Rosaceae family, native to southwestern China (Vilanova et al., 2001; Wang et al., 2017). Owing to its juiciness and nutrient-rich nature, this fruit is not only used for fresh consumption, but also in food industries for the production of juices, wines, jams, and syrups, as well as traditional Chinese medicine (Curi et al., 2017; Su et al., 2021; Dhiman et al., 2022). However, under natural conditions, loquat fruit bears among 1–5 large seeds, occupying 20–30% of fruit volume (Sadamatsu et al., 2004; Mesejo et al., 2010), which affects its palatability and processing efficiency. To produce seedless loquat, breeders have made enormous efforts. Since the 1990s, breeding triploids has been one of the main methods to produce seedless loquats and several triploid seedless varieties have been released (Guo et al., 2016; Dang et al., 2019a, Dang et al., 2019b). However, to improve the fruit set of triploid loquat varieties, hormone treatments are necessary (Badenes et al., 2013), which is both time-consuming and labor-intensive (Ramakrishnan et al., 2025). Recently, diploid loquat H30-6, a descendant of white-fleshed variety ‘Huabai 1’, has been found to bear fruits with few seeds and is an important breeding material with low male fertility (Xia et al., 2022). This germplasm serves as a crucial resource for breeding seedless loquats and studying the seedless and male sterile mechanisms.

Our previous study has reported that the low male fertility of H30-6 is caused by abnormal development of pollen mother cells (Xia et al., 2022). Various factors are associated to the development of pollen, including transcription factors (Wang et al., 2023), non-coding RNAs (Nie et al., 2023), reactive oxygen species (Liu et al., 2022) and plant hormones (Marciniak and Przedniczek, 2019; Luo et al., 2022). Among the plant hormones, jasmonic acid (JA) plays an essential role in stamen development and function. In *Arabidopsis thaliana*, JA biosynthesis and signaling are required in stamen development (Acosta and Przybyl, 2019; He et al., 2021; Huang et al., 2022). Numerous researches have declared that the JA biosynthetic mutants, including *opr3* (Stintzi and Browse, 2000), *aos*/*dde2* (Von Malek et al., 2002), *acx1 acx5* (Schilmiller et al., 2006), *lox3 lox4* (Caldelari et al., 2011), and *gh3.10-2 jar1-11* (Delfin et al., 2022), exhibit completely or severely male sterile, as manifested by failure of stamen filament elongation, delay of anther dehiscence, and loss of pollen vitality. Besides, the stamen development and fertility of these JA biosynthetic mutants can be rescued by exogenous application of JAs (Yan et al., 2016; Huang et al., 2017). Additionally, *ZmCOI2a* and *ZmCOI2b*, encoding the F-box protein COI1, a key component of the JA signaling pathway, redundantly regulate gametophytic male fertility in maize (Qi et al., 2022). Furthermore, JA has been found to regulate stamen development in peach (Sherif et al., 2015), almond (*Amygdalus communis* L.) (Li et al., 2021), and a male-sterile somatic cybrid citrus (Jiang et al., 2023), suggesting that JA plays a conserved role in regulating stamen development in fruit crops. It is speculated that JA may play a role in pollen development in H30-6.

To investigate the molecular mechanism underlying the low male fertility of H30-6, in this study, transcriptomic analysis was performed with anther samples from three developmental stages (pre-meiosis, meiosis and mature pollen). Furthermore, weighted gene co-expression network analysis (WGCNA) was applied to identify the related co-expression gene modules with the pollen fertility. Results of this work provide new insights into the molecular mechanisms of the low male fertility of loquat H30-6 and lay a theoretical foundation for further seedless breeding in loquat.

## 2 Materials and methods

### 2.1 Plant samples

In this study, H30-6 with a small seed quantity and two varieties ‘Huabai 1’ (HB) and ‘Jinhua 1’ (JH) with large seed quantities were used as materials. Since HB is the parent of H30-6, it was used as a control with close genetic distance. JH, with orange-flesh, differs from H30-6 in flesh color, serving as a control with distant genetic distance. All the materials were obtained from the fruit germplasm resources of the Key Fruit Lab, of the College of Horticulture and Landscape Architecture, Southwest University. According to the classification criteria of Xia et al (Xia et al., 2022). for flower buds: flower buds of H30-6 and JH with a diameter of less than 3 mm and those of HB with a diameter of less than 2 mm can be considered as having microspore mother cells in the anthers undergoing prophase of meiosis (pre-meiotic anthers); the flower buds of H30-6 and JH with a diameter of 3 to 4 mm and those of H411 with a diameter of 2 to 3 mm can be regarded as the microspore mother cells in the anthers being in the meiosis stage (meiotic anthers); flower buds of H30-6 and JH with diameters greater than 4 mm, and those of HB with diameters greater than 3 mm, possess mature anthers. Pre-meiotic, meiotic and mature anthers were collected from H30-6, HB and JH. Three replicates were set for each sample, and each replicate included 10 inflorescences. The separated stamens were frozen in liquid nitrogen for 15 min and stored in a refrigerator at -80°C for subsequent analysis.

### 2.2 RNA-seq

Total RNA was extracted using the BASY spin Plant Total RNA Extraction Kit (RN90, Aidlab Biotechnologies Co.,Ltd) according to the manufacturer’s instructions. RNA purity and RNA content were examined using the Agilent 2100 Bioanalyzer. The mRNA in the total RNA was enriched and purified by Oligo (dT) magnetic bead method, and then the mRNA was interrupted to 200-300 bp fragment. The first cDNA strand was synthesized with 6-base random primers (N6) and reverse transcriptase (M-MLV). Sequencing library construction and sequencing were performed by Illumina NovaSeq X Plus (PE 150) platform (Personalbio, Shanghai, China). After removing the adaptors and low-quality reads, the clean reads were then aligned to the loquat reference genome (Jiang et al., 2020) using HISAT2 (Kim et al., 2015). To estimate the expression levels of genes, the read counts for each gene were calculated by HTSeq (Anders et al., 2015), and then normalized to fragments per kilobase million (FPKM) mapped reads. Differentially expressed genes were identified using the fold change factor (|Log_2_(FC)| > 1) and the adjusted p-value threshold (*p* < 0.05).

### 2.3 Valication by real-time quantitative PCR

qRT-PCR was used to verify the RNA-seq results using the same samples for RNA-seq. Gene-specific primers, designed on the Primer3Plus website (https://www.primer3plus.com/), were list in **Supplementary Table S1**. qTOWER3 G touch (Analytic Jena, German) was used to perform qRT-PCR. *EjActin*, as a housekeeping gene, was used to normalize the amplification. The relative expressions of genes were calculated using the 2^-ΔΔCt^ method (Wang et al., 2022).

### 2.4 WGCNA

According to Langfelder and Horvath (Langfelder and Horvath, 2008), WGCNA was performed using R (4.3.1) with the default parameters and the soft-thresholding power (β) of 8. The top 75% of genes with the highest median absolute deviation in each sample were filtered for subsequent calculations. Based on the correlations between genes (Pearson’s correlation tests), genes were clustered into different modules. Additionally, the relationship between each module and sample was calculated (Pearson’s correlation tests) to construct topological overlap matrix. The top hub gene in each module was selected based on the highest kME (eigengene connectivity) value. Moreover, Cytoscape (3.10.3) was used for the co-expression network (Shannon et al., 2003).

### 2.5 UPLC-MS/MS analysis of JA and its derivatives

The flower buds with immature anthers of H30-6 and HB were collected to detect the contents of JA and its derivatives using ultra-performance liquid chromatography (UPLC: Agilent 1290) coupled with tandem mass spectrometer (MS) system (MS/MS: Applied Biosystems 6500 Quadrupole Trap). The samples were ground into powder in liquid nitrogen. Next, 50 mg of the powder was dissolved in 0.5 mL of a mixture of isopropyl alcohol, water, and hydrochloric acid (volume ratio of 2:1:0.002), and shaken at 100 r/min on a shaker at 4°C for 30 minutes. Then, 1 mL of dichloromethane was added to the sample extract and shaken for another 30 minutes. After centrifuging at 13,000 × g for 5 min, the lower layer solution of the extract was dried with a nitrogen blower. Subsequently, the sample was dissolved in 0.1 mL of methanol solution, centrifuged at 13,000 g for 5 min, and the supernatant was taken for UPLC-MS/MS analysis. The standard solutions of JA, MEJA, 12-OPDA, and JA-ILE with gradients of 0.1, 0.2, 0.5, 2, 5, 20, 50, and 200 ng/mL were prepared using methanol (0.1% formic acid) as the solvent.

The chromatographic conditions were as follows: 2.7 µm, 2.1 × 150 mm (Agilent Poroshell 120 SB-C18). The column temperature was set to 40°C. The mobile phase consisted of solvent A, methanol containing 0.1% formic acid, and solvent B, 0.1% formic acid aqueous solution. The detection adopted a gradient elution program: 20% A and 80% B for 3 min, 50% A and 50% B for 6 min, 90% A and 10% B for 1.6 min, 20% A and 80% B for 2.9 min. The injection volume was 2 µL, and the flow rate was 0.2 mL/min.

The mass spectrometry conditions were as follows: electrospray ionization source temperature, 400°C; ionspray voltage, 4500 V; curtain gas, ion source gas I, and gas II set at 15, 65, and 70 psi, respectively; monitoring mode, multiple reaction monitoring. In the Q-Trap6500 system, each ion pair was scanned based on the optimized declustering potential and collision energy. Analyst software (v 1.6.3) was used to process mass spectrometry data.

### 2.6 Observation of pollen quantity and pollen germination

According to the previous study (Park et al., 2002), the inflorescences of H30-6, containing microspore mother cells in the pre-meiotic stage, were sprayed with 2 mM methyl jasmonate (MeJA) solution or mock (water) every week until the microspore mother cells entered the post-meiosis stage (approximately one month). When blossom, pollen quantity and pollen germination of H30-6, treated with MeJA and mock, and HB were detected. Fifteen dried anthers were crushed using tweezers in 200 μL of BK medium, containing 150 g/L sucrose, 100 mg/L boric acid and 1 g/L agar, with 6 replications. Pollen quantity was counted in a blood counting chamber. Pollen germination was tested according to our previous study (Xia et al., 2022). The dried anthers were placed in 200 μL of BK medium and shaken slightly to disperse the pollen. Then, the anthers were removed. After 45 min of incubation at room temperature, 50 μL of pollen suspension was transferred into a new 1.5 mL centrifuge tube and incubated upside down for 4 h at 25°C. After being shaken, 15 μL of pollen suspension was dropped on a glass slide for the observation and imaging using an Olympus (BX35) fluorescence microscope (Tokyo, Japan). Six replicates were set for each sample. At least three photographs of different fields of view were taken per replicate and germinated and total pollen were counted.

### 2.7 Statistical analysis

SPSS (v22) software was used to conduct statistical analysis. To determine statistical significance among groups, one-way analysis of variance (ANOVA) was adopted when the variances were homogeneous; otherwise, the Welch’s test was selected. For differences between two groups, the independent-samples *t*-test was used.

## 3 Results

### 3.1 The number of seeds in the fruits of HB, JH, and H30-6

**Figure 1:**
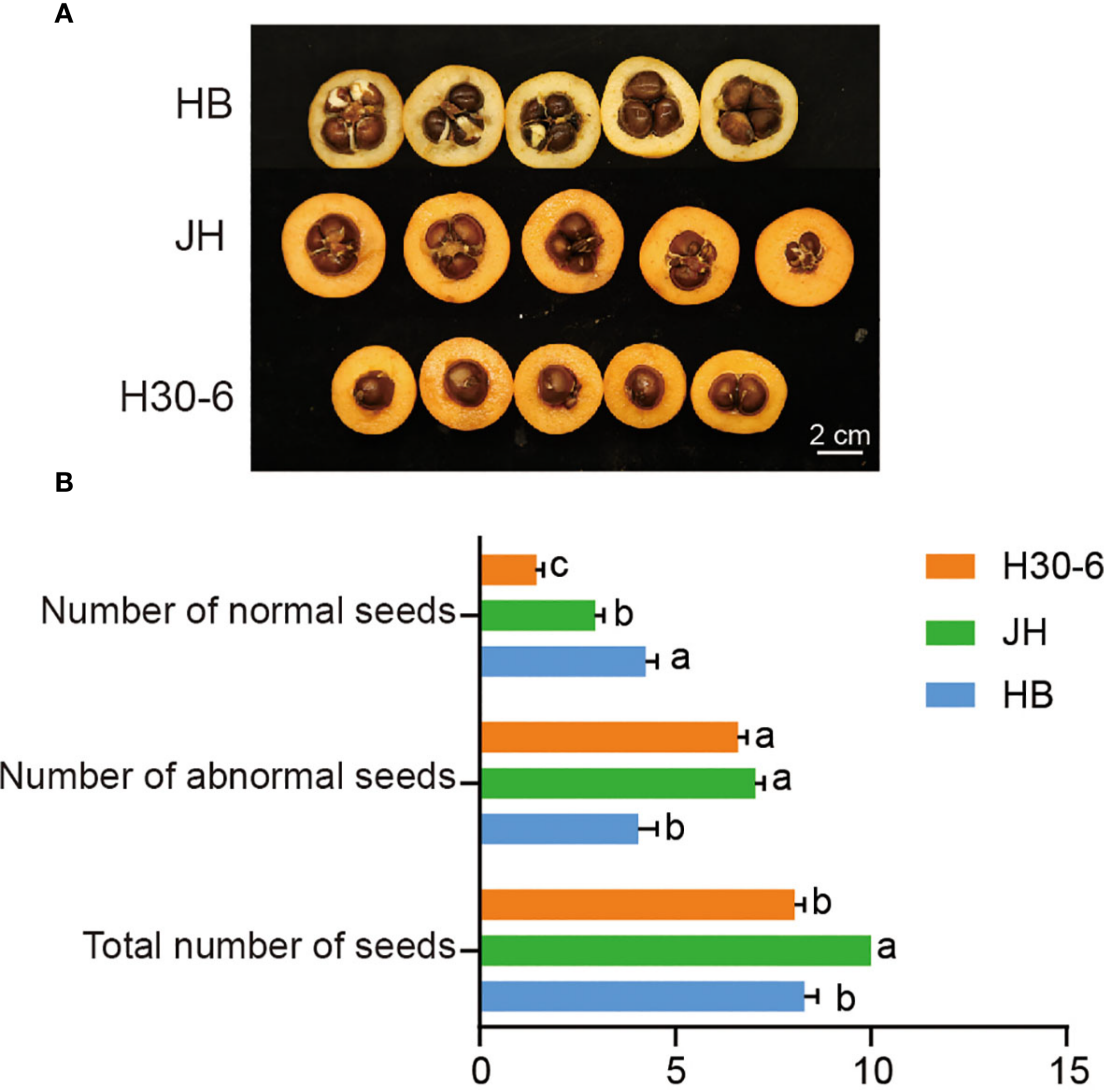
The number of seeds in the fruits of ‘Huabai 1’ (HB), ‘Jinhua 1’ (JH), and H30-6. **(A)** Cross-sections of the fruits of HB, JH, and H30-6. **(B)** The normal-, abnormal-, and total seed quantities of HB, JH, and H30-6. Lower case letters indicate significant differences in number of normal seeds, number of abnormal seeds, and total number of seeds, (*p* < 0.05, ANOVA and Tukey’s test), with 20 replicates. Error bars represent standard error (SE).

Mature fruits of HB, JH, and H30-6 were collected to count their seeds (**Figure 1A**). For the number of normal seeds, HB had the most seed quantities with an average of 4.25 seeds per fruit, significantly higher than the average of 2.95 seeds per fruit for JH and 1.45 seeds per fruit for H30-6 (*p* < 0.05) (**Figure 1B**). However, the numbers of abnormal seeds (longitudinal diameter < 1 cm) of H30-6 (**Supplementary Figure S1**) and JH were higher than that of HB (*p* < 0.05). Besides, there was no difference in the total number of seeds, including normal and abnormal seeds, between H30-6 and HB (**Figure 1B**). The number of seeds mentioned below refers to the quantity of normal seeds.

### 3.2 RNA-seq and identification of DEGs

**Figure 2:**
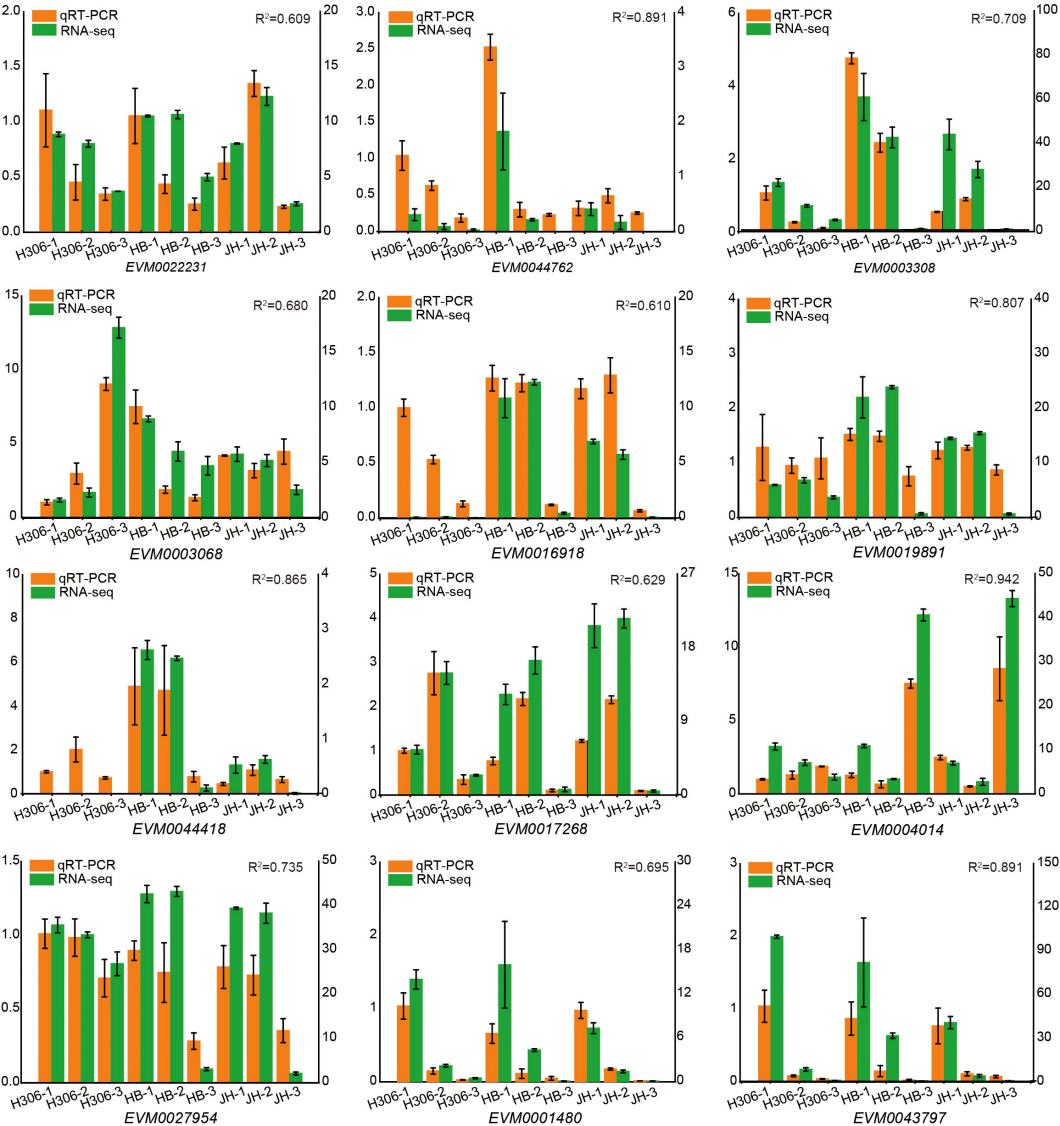
qRT-PCR validation of expression levels of 12 DEGs identified by RNA-seq. The left vertical axis stands for relative expression of genes (orange column chart) measured by the qRT-PCR. The right vertical axis stands for fragments per kilo-base per million mapped reads (FPKM) for RNA-seq results. Error bars represent SE with three replicates. R^2^ indicates coefficient of determination between qRT-PCR results and FPKM values calculated using SPSS 22.0. H306-1, HB-1 and JH-1 represent the pre-meiotic anthers of H30-6, HB, and JH; H306-2, HB-2 and JH-2 represent the meiotic anthers of H30-6, HB, and JH; H306-3, HB-3 and JH-3 represent the mature anthers of H30-6, HB, and JH.

The low male fertility of H30-6 pollen is a limiting factor for the fruit setting (Xia et al., 2022). To reveal the molecular mechanism underlying the low male fertility of H30-6, the RNA-seq was performed with pre-meiotic, meiotic and mature anthers collected from H30-6, HB and JH. For the sample names of H30-6, HB and JH, the pre-meiotic anthers were named H306-1, HB-1 and JH-1; the meiotic anthers were named H306-2, HB-2 and JH-2; similarly, the mature anthers were named H306-3, HB-3 and JH-3. Using the Illumina platform, an average of about 42 million clean reads were generated per sample. The average mapping rate was 96.03% after aligning to the loquat reference genome. A total of 45,743 genes were detected, of which 31,780 genes were expressed (FPKM > 1) in at least one sample and 12,611 were expressed in all samples. A total of 12 genes were selected randomly to perform qRT-PCR for verifying the RNA-seq results. The results showed that the relative expression trends of these 12 genes were consistent with the RNA-seq results, and the determination coefficients (R^2^) were all greater than 0.6, supporting the reliability of the RNA-seq results (**Figure 2**).

**Figure 3:**
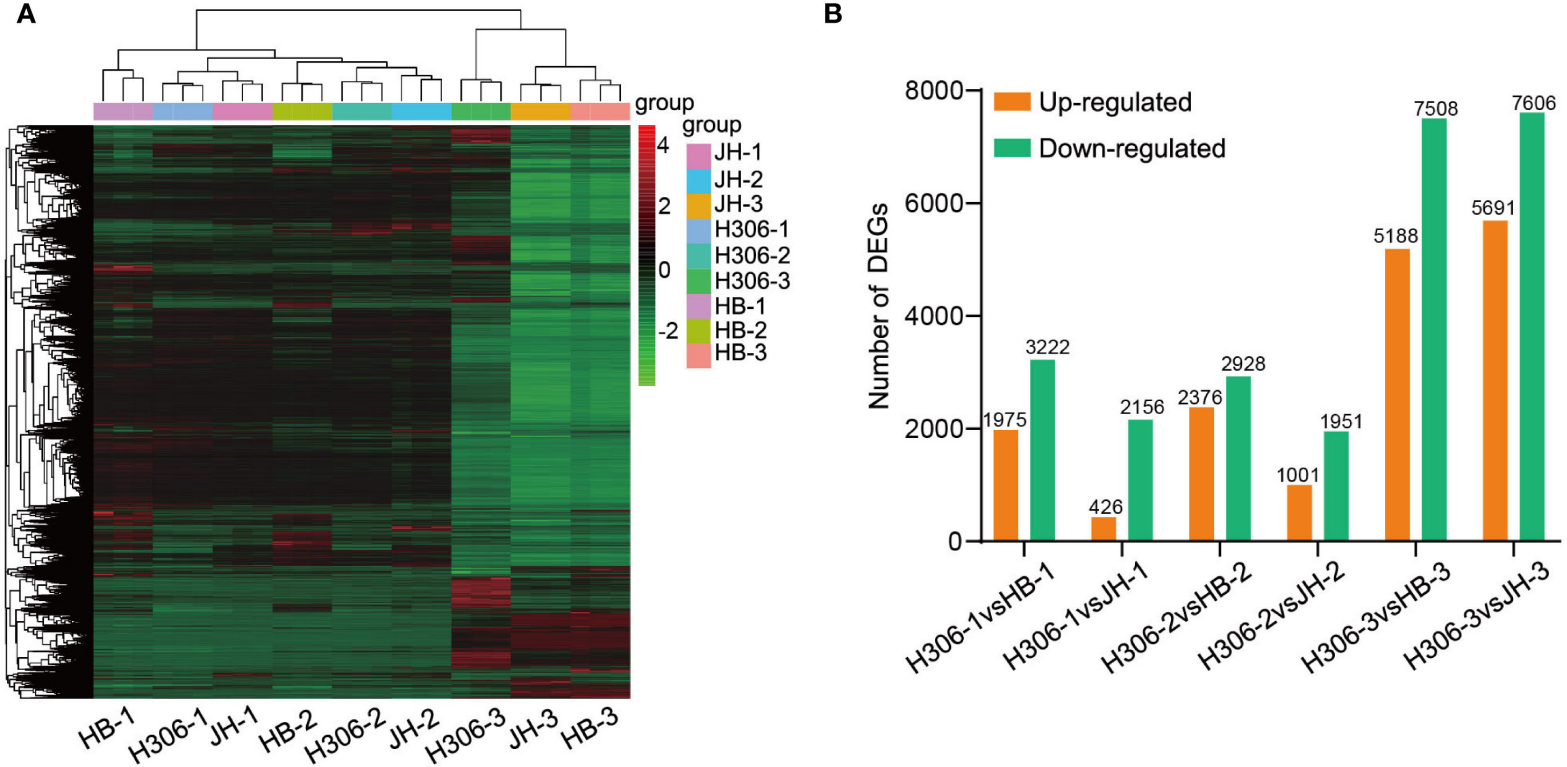
Landscape of RNA-se results. **(A)** Hierarchical clustering of differentially expressed genes (DEGs) using Eucledian distance and complete linkage methods. **(B)** The number of DEGs in H30-6 compared to HB and JH.

To evaluate the expression patterns of DEGs among samples, hierarchical clustering analysis were conducted. According to the heatmap, DEGs were clustered separately by period and clearly divided into two groups, namely mature and immature anther groups (**Figure 3A**). Compared with HB and JH, the number of up-regulated genes in H30-6 was lower than that of down-regulated genes at different periods (**Figure 3B**). Besides, the DEG number in mature anther group was higher than that in immature anther group in this comparison. For pre-meiotic anthers, 1,975 and 426 genes were up-regulated and 3,222 and 2,256 genes were down-regulated in H306-1 by comparing with HB-1 and JH-1, respectively. Regarding the meiotic anthers, there were 2,376 and 1,001 up-regulated genes and 2,928 and 1,951 down-regulated genes in H306-2 in comparison with HB-2 and JH-2, respectively. By comparison to HB-3 and JH-3, 5,188 and 5,691 genes were up-regulated, and 7,508 and 7,606 genes were down-regulated in H306-3 (**Figure 3B**).

### 3.3 The function analysis of DEGs in immature anthers

**Figure 4:**
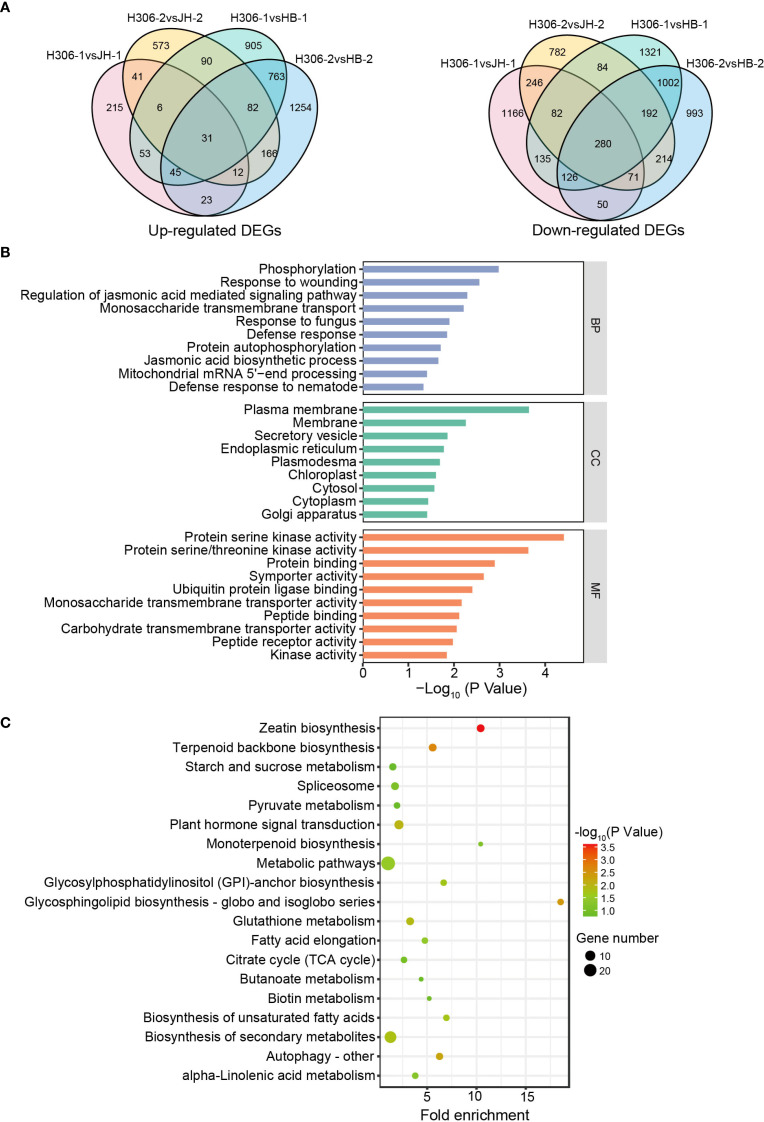
The function analysis of DEGs in immature anthers. **(A)** Venn diagrams of up- and down-regulated DEGs in DEGs in H30-6 compared to HB and JH. **(B, C)** GO term **(B)** and KEGG pathway **(C)** enrichment analyses of the 311 shared DEGs.

Since the male sterile of H30-6 is attributed to abnormal meiotic synapses (Xia et al., 2022), the pre-meiosis and meiosis phases of microspore mother cells are critical for the pollen development in H30-6. In pre-meiotic and meiotic anthers, 31 up-regulated DEGs and 280 down-regulated DEGs were shared in the comparisons between H30-6 and HB and between H30-6 and JH. (**Figure 4A**). To explore the function of the 311 shared DEGs, Gene Ontology term (GO) and Kyoto Encyclopedia of Genes and Genomes (KEGG) pathway enrichment analyses were performed. For GO enrichment analysis, these genes were enriched in biological process (BP), cell component (CC), and molecular function (MF), respectively. BP terms were mainly enriched for genes related to JA, including regulation of JA mediated signaling pathway and JA biosynthetic process. CC terms were predominantly enriched for genes associated with plasma membrane and MF terms were primarily enriched for genes involved in protein serine kinase activity (**Figure 4B**). KEGG enrichment analysis indicated that the 311 shared DEGs were mainly enriched in zeatin biosynthesis and glycosphingolipid biosynthesis-globo and isoglobo series (**Figure 4C**). Zeatin is a precursors to the synthesis of cytokinin, which ooperates with JA to regulate stamen development (Song et al., 2013; Yu et al., 2019). Glycosphingolipids, a type of sphingolipid, are key components of the cell wall, and JA is involved in the metabolism of sphingolipid (Huang et al., 2021). These findings indicate that JA may participate in the mechanism of the low male fertility of H30-6. Among the JA-related DEGs, compared with HB, *EjAOS3* (*EVM0010750*) had the lowest log_2_(fold change) values in pre-meiotic and meiotic anthers of H30-6, which were -4.32 and -5.50, respectively, followed by *EjAOC4.1* (*EVM0005305*), which were -2.78 and -5.03, respectively.

### 3.4 Weighted gene co-expression network analysis

**Figure 5:**
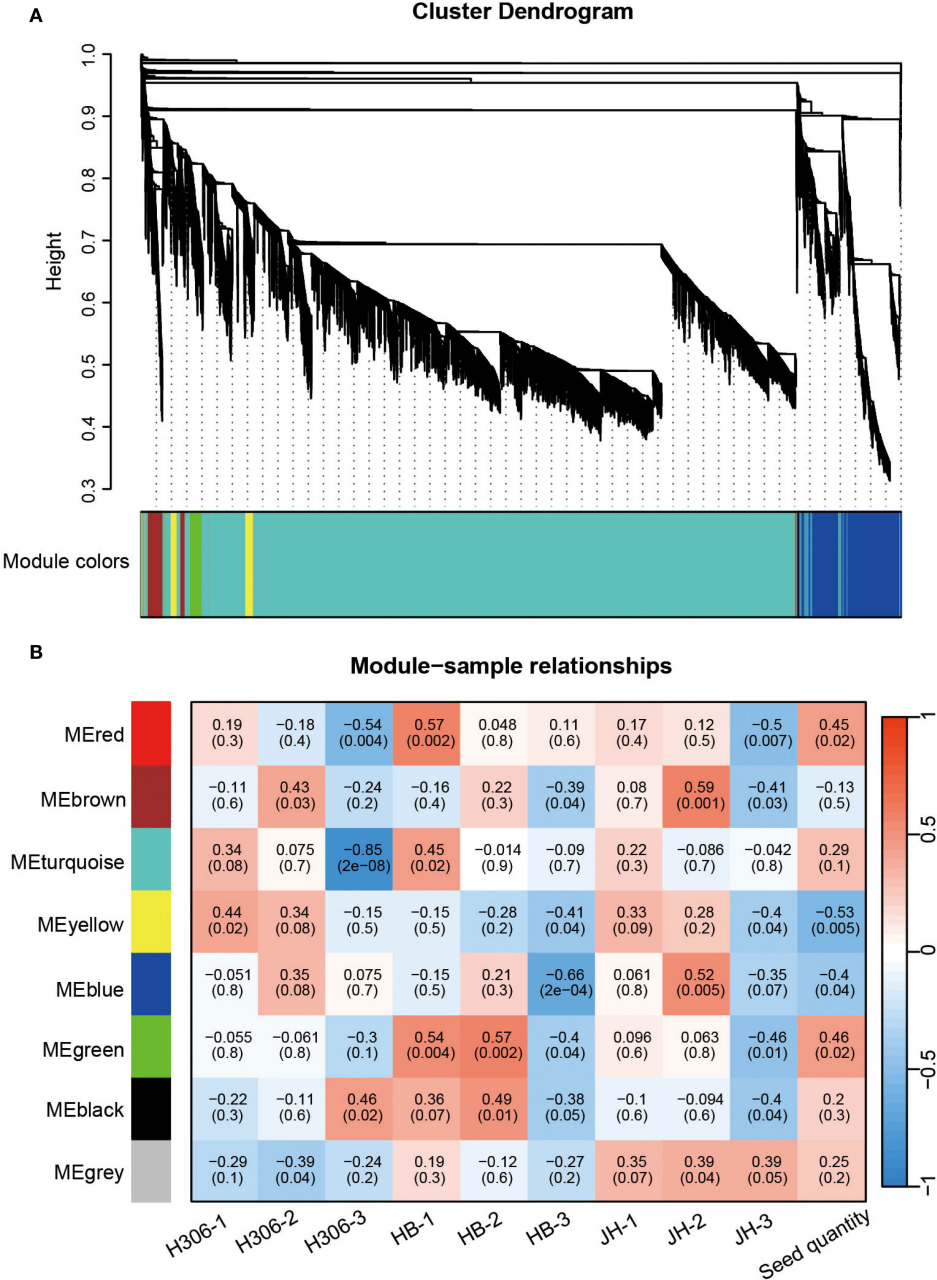
Weighted gene co-expression network analysis (WGCNA). **(A)** The cluster dendrogram and color display of co-expression network modules. **(B)** Heatmap of the correlation between module eigengenes and samples.

**Figure 6:**
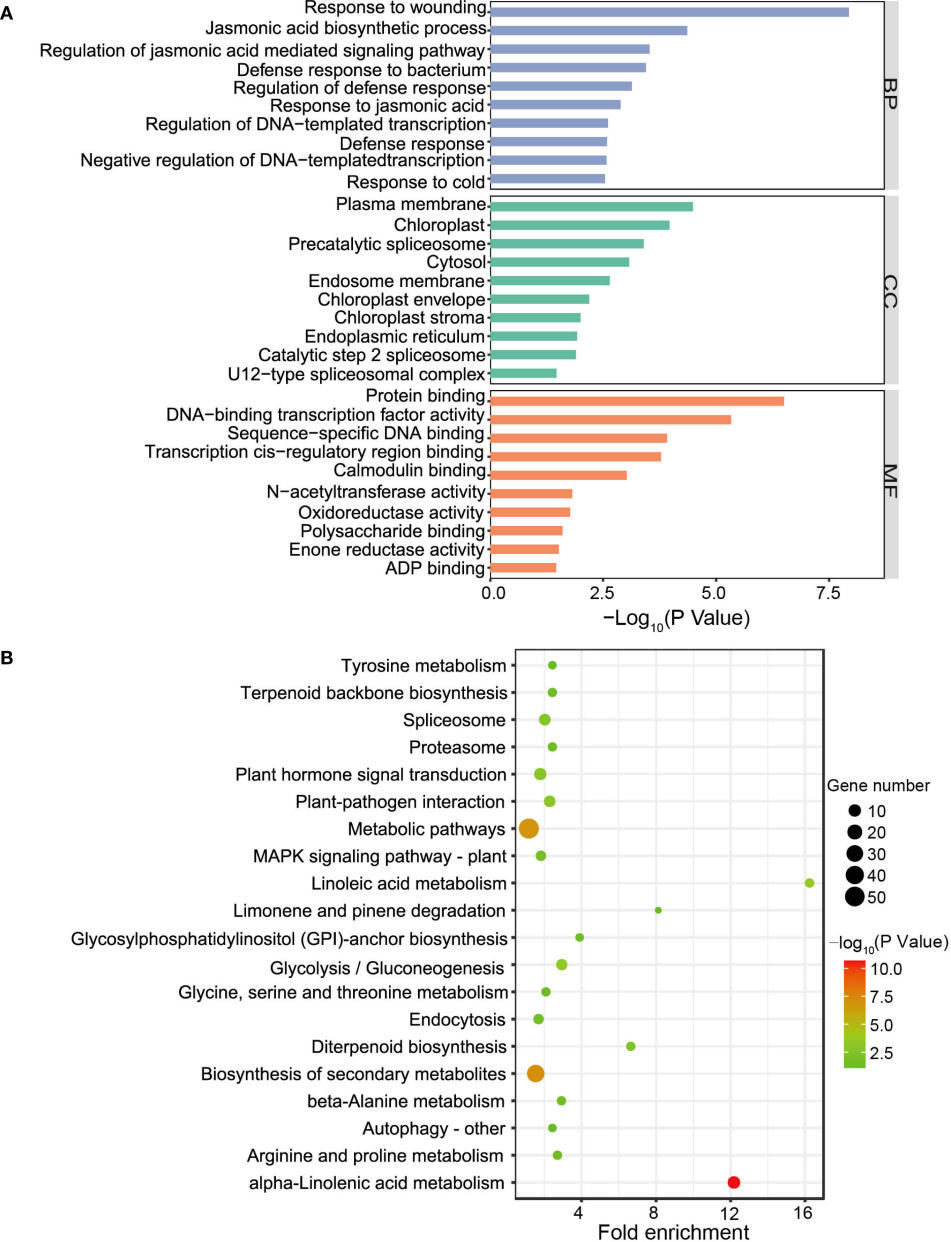
The function analysis of co-expression genes in ‘green’ module. **(A, B)** GO term **(A)** and KEGG pathway **(B)** enrichment analyses of the co-expression genes in ‘green’ module.

To investigate the relationship between gene networks with the low male fertility of H30-6, WGCNA was performed to construct the co-expressed gene modules with a total of 22,779 expressed genes. All these genes were clustered into 8 modules (**Figure 5A**). The correlation between gene modules and samples revealed that module ‘green’ was highly related with HB-2 (*r* = 0.57) and HB-1 (*r* = 0.54), and subsequently with JH-1 (*r* = 0.096) and JH-2 (*r* = 0.063) (**Figure 5B**), indicating that module ‘green’ may be related to the meiosis behavior of their pollen mother cells. Besides, module ‘green’ were highly positively correlated with seed quantity (*r* = 0.46) (**Figure 5B**), suggesting that module ‘green’ is the candidate module associated with loquat seed number. GO term and KEGG pathway enrichment analyses were performed to explore the function of the genes in module ‘green’, a total of 398 genes. For GO enrichment analysis, BP terms were also primarily enriched for genes related to JA, including JA biosynthetic process, regulation of JA mediated signaling pathway and response to JA. CC terms were mainly enriched for genes associated with plasma membrane and MF terms were predominantly enriched for genes involved in protein binding (**Figure 6A**). Besides, KEGG enrichment revealed that genes in module ‘green’ were enriched in alpha-linolenic acid metabolism (**Figure 6B**), which is the previous step of JA biosynthesis. These results further suggest that JA play an important role in the molecular mechanism underlying the low male fertility of H30-6.

### 3.5 Networks of genes related to JA biosynthesis and signaling pathway

**Figure 7:**
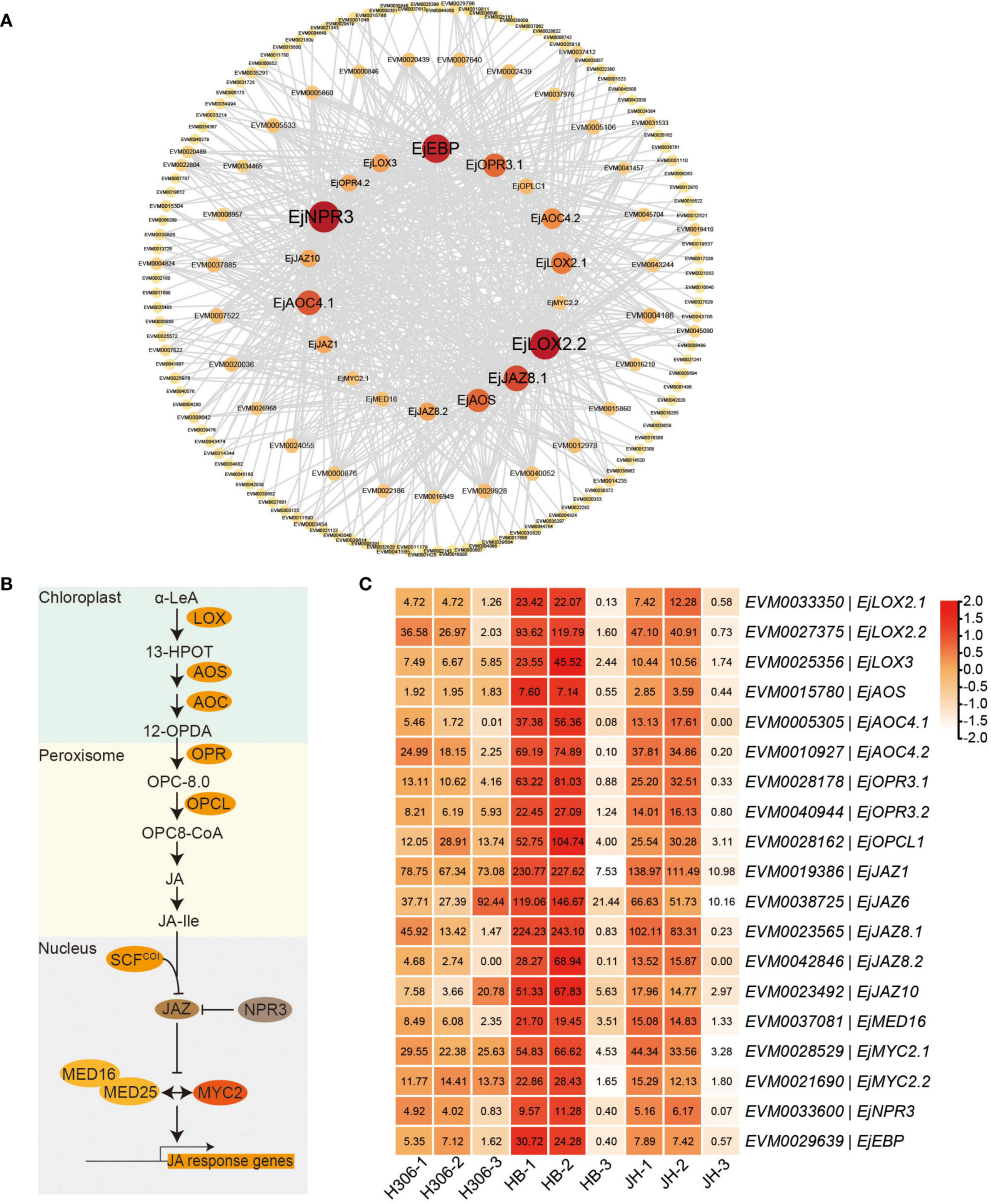
Genes related to JA in the ‘green’ module**(A)** Interaction network of genes co-expressed with the genes of JA biosynthesis and signaling pathways in the ‘green’ module. The inner circle is composed of the genes of JA biosynthesis and signaling pathways. The middle circle and the outer circle represent genes with degrees ranging from 10 to 18 and below 10, respectively. **(B)** Schematic overview of JA biosynthesis and signaling pathway from α-linolenic acid (α-LeA). **(C)** Expression heatmap of the genes related to JA biosynthesis and signaling pathway in the ‘green’ module. LOX, lipoxygenase; AOS, allene oxide synthase; AOC, allene oxide cyclase; OPR, 12-oxophytodienoate reductase; OPCL, OPC8-CoA ligase; JAZ, jasmonate-ZIM domain; NPR, non‐expressor of pathogenesis‐related protein; MED, mediator; EBP, ethylene-responsive element binding protein.

**Figure 8:**
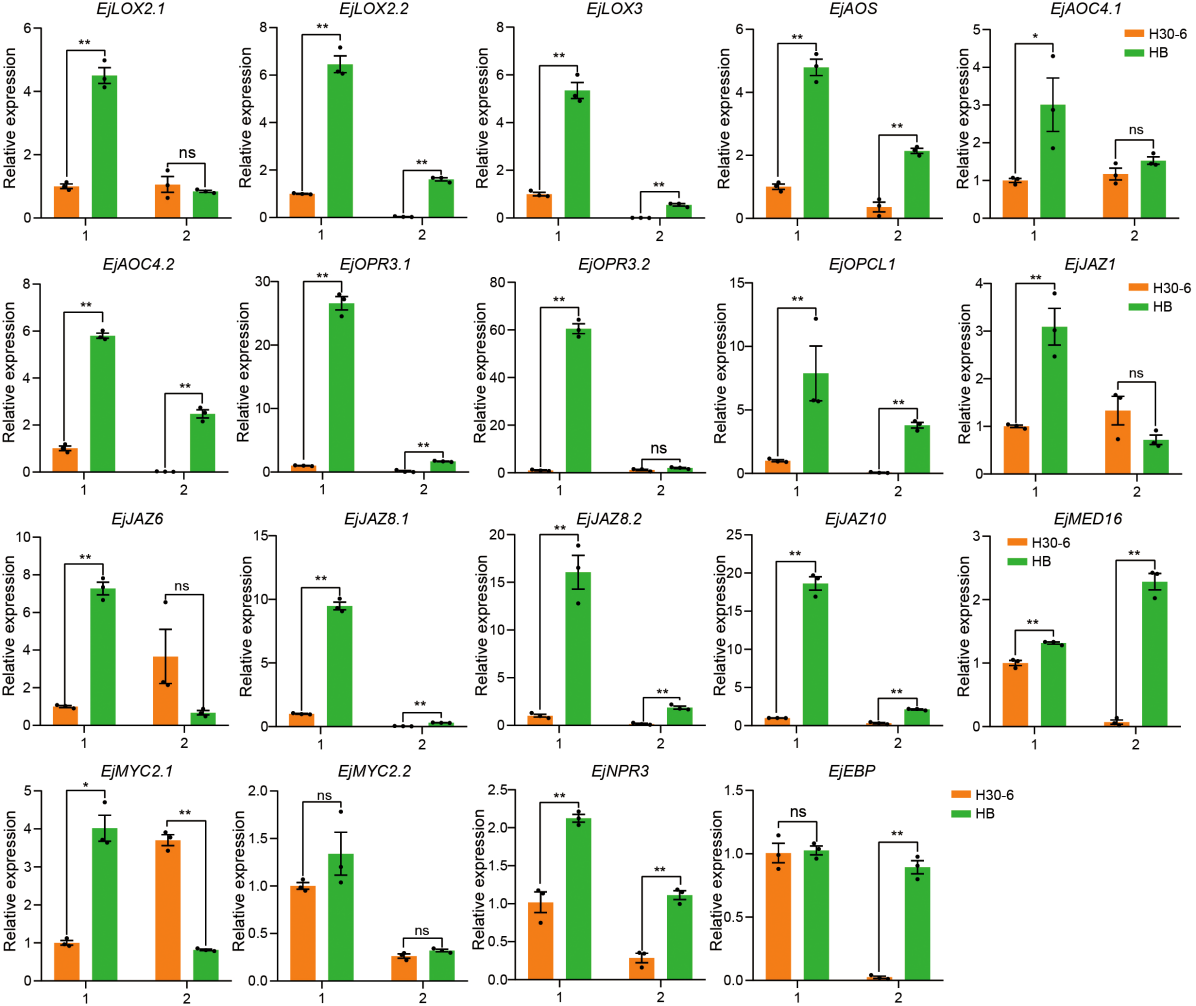
The relative expression levels of genes related to JA biosynthesis and signaling pathways in pre-meiotic (1) and meiotic (2) anthers of H30-6 (orange) and HB (green) detected by qRT-PCR. *p < 0.05; **p < 0.01 (independent samples test). Black dots present the replicates. Error bars represent SE.

To further explore the interaction between genes related to JA in the ‘green’ module, the interaction network was plotted for the co-expressed genes connected to genes of JA biosynthesis and signaling pathways with highest intramodular connectivity (KIM > 0.3) (**Figure 7A**). *EVM0033600* (*EjNPR3*) and EVM0027375 (*EjLOX2.2*) have the top two highest degrees, 76 and 72, respectively. NPR3 and LOX2 are important components of JA signaling and biosynthesis, respectively (**Figure 7B**). Expression heatmap revealed that the expressions of genes related to JA biosynthesis (*LOX*s, *AOS*, *AOC*s, *OPR*s, and *OPCL*1), JA signaling (*JAZ*s, *MED16*, *MYC2*s, and *NPR3*), and JA response (*EBP*) in both pre-meiotic and meiotic anthers of HB and JH were higher than those in their mature anthers (**Figures 7B, C**). Notably, during pre-meiotic and meiotic period, the expression of these genes in H30-6 was the lowest among the three varieties (**Figure 7C**), which was consistent with their male fertilities (Xia et al., 2022). Especially in the meiotic period, the expression levels of *EjAOC4.1* and *EjJAZ8.2* were 31.76- and 24.17-fold lower in H30-6 than in HB, respectively. Furthermore, the relative expression levels of the 19 genes related to JA biosynthesis and signaling pathways in pre-meiotic and meiotic anthers of H30-6 and HB have been verified by qRT-PCR. The results indicated that, except for *EjMYC2.2* and *EjEBP*, the remaining 17 genes in pre-meiotic anthers had significantly differences lower relative expression levels in H30-6 than HB (*p < 0.05*). Particularly, the relative expression level of *EjOPR3.2* in HB was 60 times that of H30-6. In meiotic anthers, the relative expression levels of 13 genes out of the 19 genes were significantly different between H30-6 and HB (*p < 0.05*) (**Figure 8**). These results suggested that the differential expression of genes related to JA biosynthesis and signaling pathways between H30-6 and HB in pre-meiotic and meiotic anthers may be associated with their fertility.

### 3.6 Differences in the content of JA and its derivatives in H30-6 and HB flower buds

**Figure 9:**
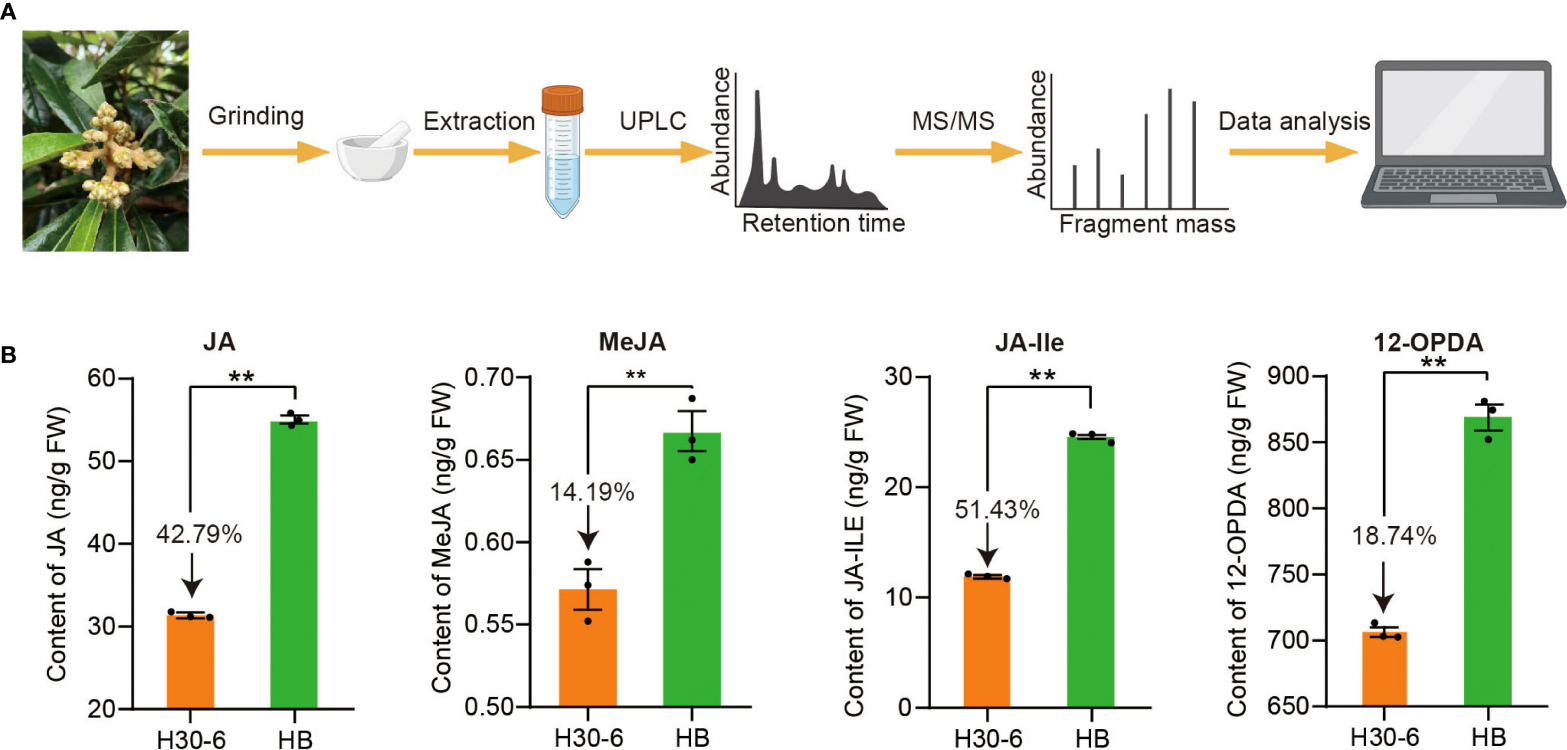
**(A)** Procedures for extraction and UPLC-MS/MS analysis of jasmonic acid (JA) and its derivatives, including methyl jasmonate (MeJA), jasmonoyl-isoleucine (JA-Ile), and 12-Oxo-phytodienoic acid (OPDA) in loquat flower buds. **(B)** The contents of JA, MeJA, JA-Ile, and 12-OPDA, in flower buds of H30-6 and HB. **p < 0.01 (independent samples test). Black dots present the replicates. Error bars represent SE.

To explore the function of JA in the pollen development of H30-6, the contents of JA and its derivatives, including methyl jasmonate (MeJA), jasmonoyl-isoleucine (JA-Ile), and 12-Oxo-phytodienoic acid (OPDA), were detected using UPLC-MS/MS in H30-6 and HB flower buds (**Figure 9A**). It was found that the contents of JA and its derivatives in H30-6 were significantly lower than those in HB (*p* < 0.01) (**Figure 9B**). The contents of JA, MeJA, JA-Ile, and 12-OPDA in H30-6 were 42.79%, 14.19%, 51.43%, and 18.74% lower than those in HB, respectively. These findings imply that the contents of JA and its derivatives in flower buds may be related to the male fertilities of H30-6 and HB.

### 3.7 Effects of MeJA on the pollen quantity and activity of H30-6

**Figure 10:**
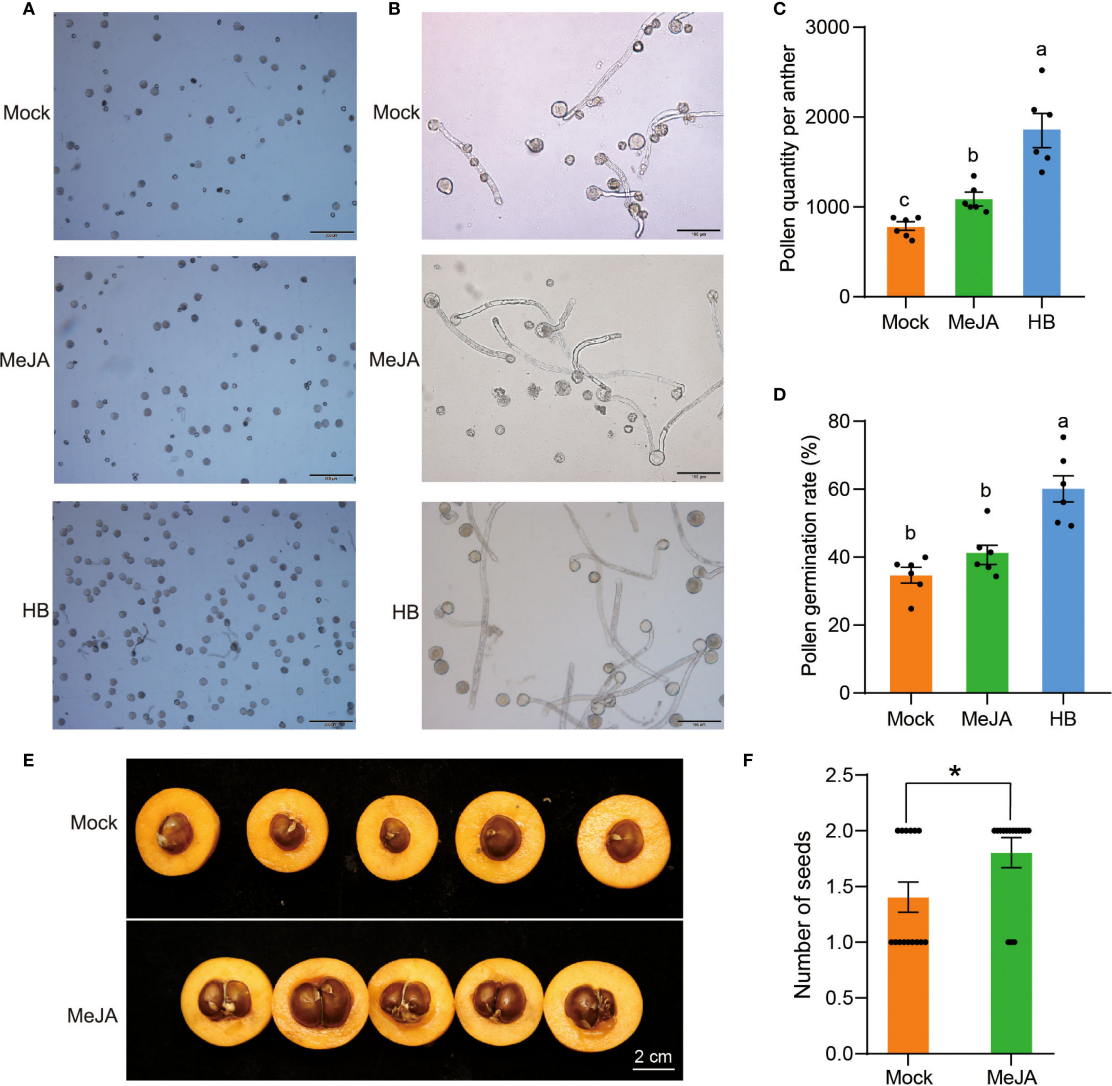
Effects of MejA on the pollen and seed number of H30-6. **(A, B)** Microscopic observation of pollen grains **(A)** and pollen germination **(B)** of H30-6 after MeJA treatment. **(C-D)** Statistics of pollen quantity **(C)** and pollen germination rate **(D)** of H30-6 after MeJA treatment. **(E, F)** Cross-sections of the fruits **(A)** and statistics of seed numbers **(B)** of H30-6 after MeJA treatment. Mock, treat with water. Lower case letters indicate significant differences in pollen quantity (*p* < 0.05, Welch’s ANOVA and Games-Howell) and pollen germination rate (*p* < 0.05, ANOVA and Tukey’s test). *p < 0.05 (independent samples test). Black dots present the replicates. Error bars represent SE.

To evaluate the effects of MeJA on the pollen quantity and activity of H30-6, the flower buds of H30-6 were sprayed weekly with MeJA, from anthers in the pre- to post-meiotic stage. Microscopic observation revealed that the numbers of pollen grains and pollen germinations of HB were obviously higher than that of H30-6 treated with MeJA and water (Mock), at the same field-of-view magnification (**Figures 10A, B**). Statistically, pollen quantity per anther of MeJA group was remarkable higher than that of Mock (water) group, but lower than that of HB (*p* < 0.05), and MeJA treatment increased pollen quantity per anther by 40.11% (**Figure 10C**). Besides, the pollen germination rate of HB was significantly higher than that of H30-6 in both MeJA and Mock groups (*p* < 0.05), while, there was no difference between MeJA group and Mock group (**Figure 10D**). These findings indicate that MeJA may improve the pollen quantity per anther of loquat.

### 3.8 Effect of MeJA on the seed quantity of H30-6

Fruits in MeJA and Mock groups were collected at maturity to count the number of seeds. Cross-sections of the fruits (**Figure 10E**) and statistics of seed numbers (**Figure 10F**) indicated that the average number of seeds per fruit in the MeJA group was 1.8, which was significantly higher than the average of 1.4 in Mock group (*p* < 0.05). MeJA treatment increased seed numbers by 28.57% (**Figure 10F**), which was lower than the increase in pollen quantity (40.11%) after MeJA treatment. It suggests that, in addition to pollen quantity, there may be other factors that contribute to the low seed yield of H30-6, such as gynoecium size and ovule number (Cucinotta et al., 2020; Yu et al., 2022). These results imply that MeJA has a positive effect on seed quantity of H30-6.

## 4 Discussion

### 4.1 Meiosis abnormality is the key factor contributing to H30-6 male sterility

Male sterility is an important breeding material to product seedless fruits in fruit trees and vegetables, as it is able to reduce costs and improve seed purity (Cheng et al., 2022; Zhou et al., 2023; Fan et al., 2025b). In plants, male sterility usually manifests as an inability to produce functional pollen or abnormal anther development, resulting in an inability to pollinate and reproduce (Zhou et al., 2022). The development of anthers in flowering plants involves a highly delicate and complex process, and even slight abnormalities in this process can lead to male infertility (Cai et al., 2025). According to phenotype, forms of male sterility can be divided into tapetal cell developmental abnormality, meiotic arrest of pollen mother cells, incomplete anther development, pollen abortion, and non-functional pollen types (Chen and Liu, 2014). Our previous work has revealed that the abnormal meiosis of pollen mother cells (Xia et al., 2022), is the key factor contributing to male sterility in H30-6, and there are phenomena of reduced pollen quantity and low pollen germination rate.

Meiosis abnormality is a general cause of seedlessness in fruit crops. A 2.09-Mb fragment translocation was found to cause abnormalities during meiosis, resulting in the formation of a seedless watermelon (Tian et al., 2021). By targeting and knocking out the genes *ClREC8* and *ClSPO11*, which are essential for meiosis, seedless watermelons has been successfully generated using gene editing technology (Cao et al., 2022; Jiang et al., 2024). Abnormal meiosis led to the male sterility in the seedless hybrid of citrus (Rao et al., 2024). Additionally, somatic mutations in the gene *MER3* related to meiosis were found to cause male sterility in the seedless citrus mutant (Fan et al., 2025a). Therefore, the identification of key genes involved in meiosis will contribute to seedless breeding of loquats and other fruit crops.

### 4.2 The suppression of JA-related gene expression during anther development contributes to the low male fertility of H30-6

Our work revealed that the suppression of JA-related gene expression and low JA content in immature flower buds may be key factors in H30-6 male sterility, which is in accordance with the important function of JA in regulating stamen development (Qi et al., 2015; Huang et al., 2022; Cai et al., 2025). JA plays a core role in coordinating pollen maturation, filament elongation, and anther dehiscence (Fang et al., 2024). Numerous reports indicate that the mutation of genes in the JA pathway leads to male sterility. The homologous genes of *EjLOX3*, *EjAOS*, and *EjOPR3.2* have been found to be associated with male fertility in Arabidopsis. Four independent *lox3 lox4* double mutants were male sterile, displaying abnormal anther maturation and cracking defects (Caldelari et al., 2011). The Arabidopsis mutants of *aos* and *opr3* also exhibited severe male sterility and no seed production (Stintzi and Browse, 2000; Park et al., 2002). Since the JA pathway is conserved across different species, similar mutants in species beyond Arabidopsis have been screened, such as the rice JA-deficient mutants *cpm1/osaos1* (*coleoptile photomorphogenesis 1*) (Biswas et al., 2003), *cpm2/hebiba/osaoc* (Riemann et al., 2013), and *osjar1* (Xiao et al., 2014), exhibiting impaired anther dehiscence or abnormal anthesis resulting in partial male sterility (Qi et al., 2022). However, similar mutants have not been identified in fruit crops. Additionally, MYC2 is a core transcription factor in JA signaling and plays an essential role in plant stamen development (Li et al., 2023). MYC2/MYC4 have been reported to enhance anther dehiscence by activating the expression of *NST1* under blue light conditions (Zhang et al., 2018). NtMYC2a mediated process of pollen maturation in tobacco (*Nicotiana tabacum* L. cv. TN90) by regulating the starch metabolism in the pollen grains, anther walls and ovaries (Bian et al., 2022).

In immature flower buds of H30-6, the expression levels of genes related to JA biosynthesis and signaling pathways as well as JA content were significantly lower than those in HB and JH. These results imply that, compared with HB and JH, natural variation may exist in the JA upstream synthesis genes (e.g., *EjLOX*s) or transcription factors regulating JA synthesis such as AP2/ERF (Chen et al., 2016), NAC (Su et al., 2020), and WRKY (Javed and Gao, 2023) in H30-6. The potential reasons for the differences in JA signal sensitivity among H30-6, HB and JH need further exploration.

### 4.3 Exogenous application of MeJA increases the pollen quantity and seed numbers of H30-6

A number of studies indicated that reduced fertility caused by JA synthesis defects can be rescued by MeJA treatment (Qi et al., 2015; Yuan and Zhang, 2015). Severe male sterility was observed after knock-out of *AOS* in Arabidopsis, which was rescued by exogenous application of MeJA (Park et al., 2002). In cotton, exogenous application of MeJA to early buds of *Ghaoc2* mutant lines rescued the phenotypes of sterile pollen and indehiscent anther (Khan et al., 2020). Besides, the application of MeJA significantly upregulated the pollen fertility of photoperiod-sensitive genic male sterile rice, resulting in partial fertility and even reversing the expected natural sterility (He et al., 2021). The treatment of MeJA induced the expression of *LoMYB26*, promoting endothecium lignification and anther dehiscence in lily (Fang et al., 2025). In this study, the treatment of MeJA improved the pollen quantity per anther (**Figure 10C**) and seed numbers (**Figure 10F**) of H30-6, but not the pollen germination rate (**Figure 10D**).

Although JA plays an important role in coordinating pollen maturation, filament elongation, and anther dehiscence (Fang et al., 2024), its effect on pollen quantity remains unclear. We suppose that, on the one hand, MeJA treatment may promote the anther dehiscence of H30-6, leading to an increase in pollen quantity; on the other hand, MeJA treatment may have rescued the meiosis behavior of H30-6 pollen mother cells, thereby increasing the number of normal pollen grains. Multiple essential genes for meiosis have been identified in plants, including *ClREC8*, *ClSPO11* (Cao et al., 2022; Jiang et al., 2024) and *ClDYAD* (Wu et al., 2025) in watermelon, *MER3* in citrus (Fan et al., 2025a), and PHD transcription factor gene *PP1* in maize (Pan et al., 2025). More meiotic mutants have been characterized in rice, maize, tomato, wheat, Brassica, and Arabidopsis (Wang et al., 2021). Currently, there is limited research on the direct regulatory role of JA in pollen mother cell meiosis. MYC2, a core transcription factor in JA signaling, plays an essential role in plant stamen development by transcriptional regulation of downstream genes (Li et al., 2023). The MYB-MYC complex formed by the interaction of MYC2 with MYB21 and MYB24 cooperatively mediates stamen development and seed formation (Qi et al., 2015). Besides, MYC transcription factors redundantly mediate JA-induced expression of MYB genes (*MYB21*, *MYB24*, *MYB57*, and *MYB108*), thereby regulating stamen development (Li et al., 2023). In rice, GAMYB has been reported to mediate meiosis in pollen mother cells by regulating the expression of *bHLH142* (Ko et al., 2021). It is speculated that JA may target these crucial genes for meiosis through the MYB-MYC complex to regulate H30-6 rescue of male sterility, but further verification is still needed. Previous studies have found that MeJA affects pollen germination in a dose-dependent manner. It has been reported that 0.1 and 0.25 mM MeJA enhanced while 0.5- and 1 mM MeJA decreased the pollen germination in *Prunus armeniaca* L (Muradoğlu et al., 2010). Similarly, 0.05 mM and 0.25 mM MeJA significantly increased the total germination rate of *Pinus nigra* pollen, while 0.5 mM MeJA had no significant effect (Çetinbaş-Genç and Vardar, 2020). Therefore, the MeJA treatment concentration used (2 mM) in this study may not be suitable for loquat pollen germination, resulting in no significant improvement in pollen germination rate.

In fruit crops, the role of JA in stamen development has been studied. In peach, JA pathway has been reported to be involved in flower development and pollination (Sherif et al., 2015). JA has also been verified to promote anther development in almond (*Amygdalus communis* L.) (Li et al., 2021). In a male-sterile somatic cybrid citrus, JA and indole-3-acetic acid jointly influenced the expression of genes related to stamen development and interfered with the morphology of stamens and flowers (Jiang et al., 2023). These studies suggest conservative role of JA in regulating stamen development in fruit crops, supporting that JA takes part in the low male fertility of loquat H30-6.

## 5 Conclusion

To investigate the molecular mechanism underlying the low male fertility of H30-6, in this study, RNA-seq and WGCNA were performed. Both RNA-seq and WGCNA analyses revealed that suppression of JA-related gene expression during pollen development contributes to the low male fertility of H30-6. UPLC-MS/MS and exogenous application of MeJA also confirmed the importance of JA content in loquat pollen development. Our work suggests that JA is an essential factor in regulating male fertility in loquats, providing a theoretical basis for seedless breeding of loquats and laying the foundation for the development of stamens in fruit trees.

## Data Availability

RNA‐seq data of pre-meiotic, meiotic and mature anthers collected from three loquats have been deposited into the NCBI (National Center for Biotechnology Information) database with accessions number PRJNA1283964.
