# Supplementary material for: Combined analysis of transcriptome and WGCNA reveals the mediating role of JA in the low male fertility of loquat H30-6
Source: Front Plant Sci. 2025 Sep 12;16:1648313. doi: 10.3389/fpls.2025.1648313 (PMC12463989; doi:10.3389/fpls.2025.1648313)
Supplement: Supplementary file 1 [file Supplementaryfile1.docx]

**
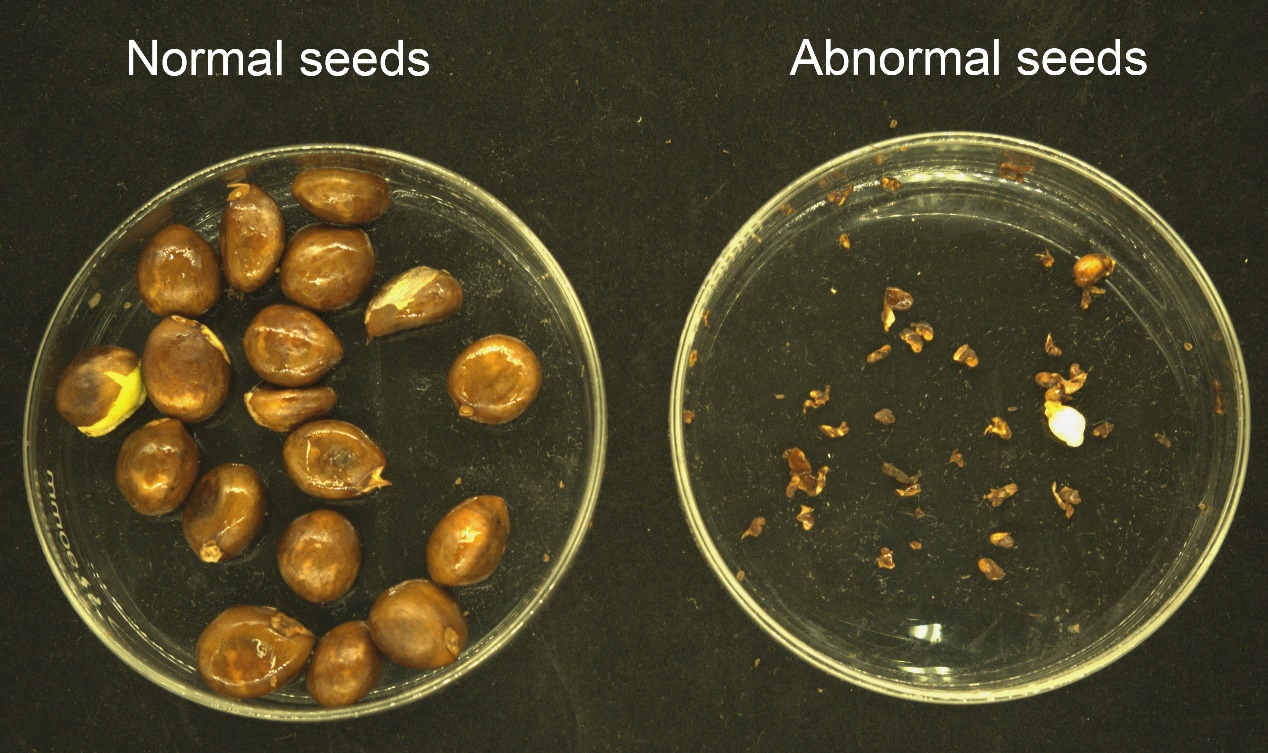
Figure S1**. The normal and abnormal seeds of H30-6.

| **Table S1 The sequence of gene-specific primers for qRT-PCR** | | |
| --- | --- | --- |
| Gene ID | F | R |
| *EjAOC* (*EVM0005305.1*) | GAGACCTTGTGCCCTTCACT | ATCTCCTTTCCTCTCCGGCT |
| *EjAOS* (*EVM0010750.1*) | GCTTGGTCACACTTTGGCTC | GACGAGAAAGGCAGGGTAGG |
| *EVM0003308.1* | CACATTGGCCCTTTCCAAACA | CATCCGCAGAGACGGAAGA |
| *EVM0019891.1* | ATGATACGATGGCCGGTGTC | TAGCAGGTTGGGTTGCTGAC |
| *EVM0016918.1* | CGCAGCGTGAGGATTTCTTT | CGCCCATTTTCACATTCCCG |
| *EVM0003068.1* | ATGTGCAAAGATGCTCATCGG | GGTAAGGGCCATCAGTTTAGAAT |
| *EVM0044418.1* | TGGCATTGTTCAGAACCGGA | TGGCACCTCCACCTGATTTC |
| *EVM0017268.1* | CCCCAAAGGGTTGGTTCACT | TGTGAGTGCAATGTTCACCG |
| *EVM0004014.1* | TCTTGAGCGAGGAGGAATGC | CAGCCTCAACTGTGAACCCT |
| *EVM0043797.1* | GGAAAGGCAAAGGCGAACAG | TCAGTCCTTCGCATCAGCAA |
| *EVM0001480.1* | GAATGGACCTCCCTCTGCAA | CCGCTCAACGTGAACAACTC |
| *EVM0027954.1* | GATCCGTTTTAAGCTTGCCGT | ACGGCGAGTATGACCTGAAG |
| *EVM0022231.1* | AAGAAAACGGAGGCCATGCTA | TGCACAGACAACGCTTCTAAC |
| *EVM0044762.1* | TCCACAACAGTGCCTCCTTC | TCTCCATTGTGATGCTTCTCCT |
| *EjLOX2.1* | AAGGGGAATGGCAGTTGAGG | TCCACCAACCTTGAAGCTCC |
| *EjLOX2.2* | AAGGGGAATGGCAGTTGAGG | TCTGCATGGCCTTTGGTTCT |
| *EjLOX3* | GCTACGTACCCATGCTACCC | ACACCATCAGCGCTGATCAA |
| *EjAOS* | GACGGGGAGAAGCTGTTGAA | ACGTCACAGTAATGGCAGCA |
| *EjAOC4.1* | GAGACCTTGTGCCCTTCACT | ATCTCCTTTCCTCTCCGGCT |
| *EjAOC4.2* | AAGCAAGTCAACTCCCTCGG | TGGCCGTAGTCTCCGAAGTA |
| *EjOPR3.1* | GGTGGATCGCTTGAAAACCG | GCTTGACCATAGGCTGCGTA |
| *EjOPR3.2* | CTCAGCCTCGTTATGCAGCT | AATCAGCATCCCCAGAAGCC |
| *EjOPCL1* | CGGACGATAACCACCACCAA | CACGGTTCCTCTGGATGGAC |
| *EjJAZ1* | ATCAAGCGATTCAGGAGGGC | GAGCAGCCAACCCTAACCAT |
| *EjJAZ6* | TCCGTCATCAAAACCCAGCA | TTTCCTTCGCCTTCTCAGCC |
| *EjJAZ8.1* | ACCCAGCAACAAGAACAGCA | GGAGACATCACAGTTGGCGA |
| *EjJAZ8.2* | CCACTTCTGCACCACCATCA | GTCCCTCCTCCTCGCATTTC |
| *EjJAZ10* | CGCCTCCTACTCCTCCAGAT | ACGTAGAGTGGCAAACAGGG |
| *EjMED16* | TCCCGAGGCTGCATTTCAAT | CGTTGCACTCTCCATCCTGT |
| *EjMYC2.1* | AGCAACGAGGAGGGGATAGT | TCAGTGGCTCTTCTCTCCCA |
| *EjMYC2.2* | TGTGAAGATAATCGGGCGGG | TCCTGCGTGTAAATTCGGCT |
| *EjNPR3* | CTTAGACGAAGCCAATGCGC | ATGGCTCTTTCCGCATCACA |
| *EjEBP* | TGCGACGTTTCCCTCCAATT | GCTCCCGTTCTGATTGCAAC |
